# Bibliographical Notices

**Published:** 1844-06-01

**Authors:** 


### BIBLIOGRAPHICAL NOTICES.

*Annual Report of the Physician and Superintendent of the Eastern Asylum, in the city of Williamsburg, Virginia, for* 1843. Richmond, 1844.

*Twenty-seventh Annual Report on the State of the Asylum for the Relief of Persons deprived of their reason.* Philadelphia, 1844.

We have on former occasions expressed the gratification we experience in common with our fellow citizens, in witnessing the number and excellence of the Institutions throughout the country for the relief of the Insane; and especially do we feel grateful for the happy results of the humane and benevolent treatment adopted in these institutions, so different from that formerly pursued, and, in too many instances, still in practice in some of the older countries.

Not a report comes to us that does not pointedly refer to this subject, and, in the language of benevolence and truth, exult in the successful triumph of the modern system. Instead of stripes and chains and the strait-jacket, rational occupation and amusements with "kindness and care, and a course of conduct springing from all the good affections;"—instead of the dungeon and the dirty cell,—the workshop, the drawing room, the lawn and the sweet parterre, are now the substitutes! And who does not rejoice at the change, even if it were followed by no better results. But experience, ample experience, has shewn that, far from restoring the shattered intellect, coercive treatment too often

——"puts out
The eye of Reason, prisons, tortures, binds,
And makes her thus, by violence and force."

The first of these reports is a pamphlet of 43 pages, well stored with facts and sensible remarks, evincing the vigilant attention of the physician and superintendent, Dr. John M. Galt, to all the concerns of the Virginia Institution, and especially his deep interest in the welfare of the afflicted subjects of insanity. We are informed by Dr. Galt that, "the number of patients who have been inmates of the Eastern Asylum since the first of January, (1843?) is one hundred and thirty five. Of these, eighty were males, and fifty-five females. Six of the male patients, and nine of the females, were coloured persons.

The number of patients on the first of January, was ninety-three, viz.: males fifty-seven, females thirty.

During the year, forty-two have been received; twenty-three of these were males, and nineteen females.

The number of the discharges is twelve, viz.: nine males and three females; one male and one female now in the institution are convalescent.

The number of the deaths is fourteen, viz.: nine males and five females; or about ten and a third per cent. The number of patients at present is one hundred and nine, viz.: males sixty-two, females forty-seven."

The other Institution, whose report we shall now briefly notice, is owned and controlled by members of the Society of Friends, mostly those of the Philadelphia Yearly Meeting. It is located about six miles from the City of Philadelphia, in the vicinity of the village of Frankford. The country around, is devoted to agriculture; the land is fertile, but high, and free from swamps, stagnant water, or other sources of malaria. The buildings are substantial and convenient, properly enclosed, and have a farm attached, from whence the Institution derives many of its supplies, as milk, butter, vegetables for the table, &c.

Originally, none were admitted as patients except members of the Society of Friends; but of late years, other respectable persons are received on the payment of a reasonable compensation, whenever the number of applicants belonging to the society is less than the institution can accommodate.

The report states: "There were forty-six patients in the Asylum, Third month 1st, 1843; forty-two have been since admitted, making a total of eighty-eight under care; being five more than last year. During the year, thirty-two were discharged, and four died. Of those discharged, seventeen were restored;—two much improved;—seven improved;—and six stationary. The number in the house on the 1st instant, (Third month, 1844?) was fifty-two, of whom three are restored;—six much improved;—five convalescent;—two improved;—and thirty-six stationary; being mostly chronic cases, long resident in the house.

The average number of patients, per the monthly enumeration, is forty-eight and six-twelfths."

Charles Evans, M. D., is the attending Physician, and Joshua H. Worthington, M. D., Resident Physician.

